# Antimitochondrial Antibody-Negative Primary Biliary Cholangitis: A Retrospective Diagnosis

**DOI:** 10.7759/cureus.36309

**Published:** 2023-03-17

**Authors:** Rami Al-Handola, Justine Chinnappan, Murtaza Hussain, Abdullahi Mahgoub, Ghassan Bachuwa

**Affiliations:** 1 Internal Medicine, Hurley Medical Center - Michigan State University, Flint, USA

**Keywords:** anti-gp210 antibody, anti-sp100 antibody, anti mitochondrial antibody, primary biliary cirrhosis, cholestatic liver injury, ama negative, primary biliary cholangitis

## Abstract

Primary biliary cholangitis (PBC) is an inflammatory cholestatic disease that tends to worsen, leading to hepatic cirrhosis and portal hypertension. We present a case of a middle-aged female who presented with progressively worsening generalized itch; the examination was significant only for urticarial rash and facial swelling. Investigation revealed direct hyperbilirubinemia, mildly elevated transaminase, and significant elevation of alkaline phosphatase. A differential was performed with labs including antimitochondrial antibodies (AMA) for PBC, hepatitis panel, anti-smooth muscle antibodies for autoimmune hepatitis, and tissue transglutaminase IgA for celiac disease, all of which were unremarkable. The patient was empirically treated with ursodeoxycholic acid (UDCA). Given the excellent clinical response at the three-week follow-up to treatment despite negative AMA, further testing with anti-sp100 and anti-gp210 was pursued, which returned positive for anti-sp100, confirming the diagnosis of PBC.

## Introduction

Primary biliary cholangitis (PBC) is a chronic progressive autoimmune inflammatory cholestatic disease that leads to biliary cirrhosis, requiring a liver transplant [1]. With varying presentations and laboratory results, observing the other uncommon variants and their presentation is essential to diagnose PBC definitively. However, delaying treatment can adversely affect patient outcomes. In this report, we present a peculiar case of a patient with antimitochondrial antibody (AMA)-negative PBC, which was later found to be anti-sp100-positive. The treatment is usually delayed for this condition, awaiting pending laboratory results, especially when AMA is negative. Early initiation of treatment with ursodeoxycholic acid (UDCA) can lead to a more rapid response and quicker remission, as seen in our patient.

## Case presentation

A 55-year-old female with a past medical history of chronic obstructive pulmonary disease, chronic back pain, gastroesophageal reflux disease, hypertension, obesity with a BMI of 41 kg/m^2^, and tobacco use disorder presented to her physician with concerns of progressively worsening generalized itch for the past four weeks. Her medication included atorvastatin, hydrochlorothiazide, lisinopril, omeprazole, an albuterol inhaler, and a fluticasone-salmeterol inhaler. The patient had visited the ER twice with the same complaint previously. The physical exam was normal at the initial presentation, and she was treated with diphenhydramine and prednisone for contact dermatitis, given her history of recent changes in laundry detergent and bath soap. She presented again four days later with diffuse urticarial rash and left-sided facial swelling with lip swelling without any signs of airway compromise. She was treated with methylprednisolone, a few doses of diphenhydramine, hydroxyzine, and tranexamic acid for likely lisinopril-induced angioedema. She was observed overnight with discontinuation of lisinopril and discharged under stable condition. Three weeks later, she presented due to progressive worsening of generalized itching not responding to prednisone, loratadine, pseudoephedrine, or diphenhydramine. She denied any change in environment or any recent change in body products, linen, or pets. She also denied abdominal pain, nausea, vomiting, melena, hematemesis, fever, weight loss, and bowel or bladder disturbances. She reported consuming alcohol socially. She denied any family or personal history of autoimmune disease.

**Figure 1:**
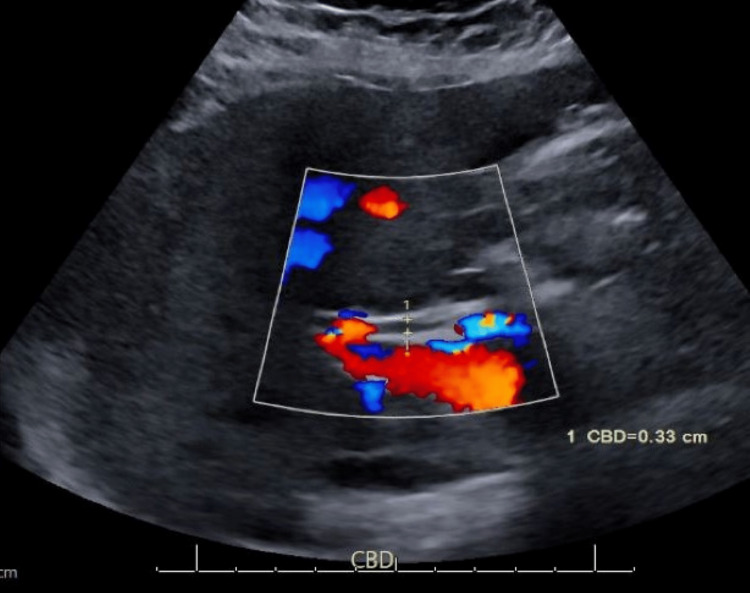
Ultrasound of the abdomen showing normal common bile duct diameter (0.33 cm)

She was hemodynamically stable and afebrile on presentation. She was distressed due to itching, and examination revealed scleral icterus, excoriation from scratch, and no skin rashes. Investigation revealed an absolute eosinophil count of 2.3 K/UL, thyroid-stimulating hormone of 0.99 UIU/ml, bilirubin total/direct of 3.3 mg/dL/2 mg/dL, aspartate aminotransferase (AST) of 187 U/L, alanine aminotransferase (ALT) of 250 U/L, and alkaline phosphatase (ALP) of 675 U/L. Given the cholestatic pattern of liver enzymes, further workup to assess for extrahepatic biliary obstruction was pursued with ultrasound, which revealed mild hepatomegaly without common bile duct dilation or cholelithiasis (Figure 1). AMA and serology testing for hepatitis B, hepatitis C, and hepatitis A virus infection were negative. As all the workup returned negative, magnetic resonance cholangiopancreatography (MRCP) was considered but the patient declined to undergo it. As the patient was extremely symptomatic to the point that it was affecting her regular daily activities, empiric treatment with prednisone 40 mg once daily for five days, UDCA 300 mg twice daily, and hydroxyzine 25 mg every eight hours as needed for itching was started. After three weeks of treatment, she had good clinical improvement both subjectively and clinically, with improved liver function, as follows - ALP: 245 U/L, AST/ALT: 39 U/L/62 U/L, total bilirubin/direct bilirubin: 0.5 mg/dL/0.2mg/dL (Table 1); there were no reported side effects due to the treatment. Given the excellent clinical response to treatment despite negative AMA, further testing with anti-sp100 and anti-gp210 was done, and the results returned positive for anti-sp100, endorsing the diagnosis of PBC. A laboratory workup done two months after the initiation of the treatment revealed complete normalization of the liver function panel. She was referred to a hepatologist for long-term follow-up. Her condition eventually stabilized as reported by her hepatologist five months later.

**Table 1:** Effects of empiric initiation of ursodeoxycholic acid

Labs (normal range)	On presentation	Three weeks after empiric treatment
Alkaline phosphatase (30-143 U/L)	675 U/L	245 U/L
Alanine transaminase (SGPT) (7-40 U/L)	250 U/L	62 U/L
Aspartate aminotransferase (SGOT) (0-40 U/L)	187 U/L	39 U/L
Total bilirubin (0.3-1.2 mg/dL)	3.3 mg/dL	0.5 mg/dL
Direct bilirubin (0.1-0.2 mg/dL)	2 mg/dL	0.2 mg/dL
Total protein (5.8-8.2 g/dL)	7.1 g/dL	6.9 g/dL
Albumin (3.3-4.8 g/dL)	4 g/dL	4.1 g/dL

## Discussion

PBC is an autoimmune disorder affecting the intralobular bile ducts with an incidence and prevalence of 1.76 and 14.60 per 100,000 people, respectively [2]. It is primarily an incidental diagnosis in this era of routine testing for liver function. When symptomatic, fatigue and pruritus are the typical presentations, followed by jaundice, as seen in our patient. In the absence of extrahepatic biliary obstruction, PBC is diagnosed by the presence of at least two of the three following criteria: elevated ALP of more than one and a half times the upper limit of normal (ULN), the presence of AMA or PBC-specific autoantibodies like gp120 and sp100 if AMA is negative, and histopathology findings of PBC [1,3]. Early diagnosis is essential for halting the progression and complications of PBC, especially due to the rapid progression of the disease if left untreated. AMA is found in approximately 95% of patients with PBC. A strong suspicion in the remaining 5% should prompt further testing for other PBC-specific autoantibodies to establish early diagnosis and treatment. The specificity of anti-sp100 and anti-gp210 antibodies are 100% and 96.5%, respectively, while they have a sensitivity of 28.3% and 29.2%, respectively. The positive and negative predictive values of the anti-sp100 antibodies were 100% and 43.3%, respectively [4].

Various studies have compared AMA-negative and AMA-positive PBC. In a Japanese study, AMA-negative PBC was found to be highly associated with complicated autoimmune disease [5]. Another study, which examined anti-sp100 and anti-gp210 antibodies in PBC, stated that both were associated with disease progression irrespective of the presence or absence of AMA [6]. A retrospective study conducted at the Mayo Clinic demonstrated significantly worse outcomes in AMA-negative PBC patients compared to AMA-positive PBC patients in terms of progression to cirrhosis, variceal bleeding, and death [7]. Another study has stated that UDCA is the initial treatment of choice for AMA-negative PBC once the diagnosis has been established. In our patient, given the unexplained progressive severe itching impairing her quality of life, jaundice, elevated ALP, and absence of extrahepatic obstruction, we had strong suspicion for PBC, prompting us to treat the patient empirically with UDCA even with a negative AMA. She had an excellent clinical response to the treatment within three weeks, which persuaded us to establish a definitive diagnosis with further testing. We deviated from the protocol of confirming the diagnosis before starting the treatment as there was a rapid progression of symptoms and elevation of liver enzymes. Following treatment with UDCA, if AST is less than three times ULN, ALP is less than three times ULN, and bilirubin is ≤1 mg/dL, the survival rate at 10 years without transplantation is 90% [8]. Having started the treatment at an early stage with an excellent biochemical response, we expect a good prognosis for her. In resource-limited settings, with negative AMA, starting the treatment with UDCA based on high clinical suspicion and biochemical follow-up will help establish the diagnosis retrospectively as well as halt disease progression, resulting in a good prognosis.

## Conclusions

This case report highlights the fact that 5% of PBC cases are AMA-negative. In cases with a high index of suspicion and negative AMA, empiric treatment with UDCA followed by testing of other antibodies specific for PBC, such as anti-Sp100 and anti-Gp210, can be performed in resource-limited settings. AMA-negative PBC has a worse prognosis when compared to AMA-positive PBC.
